# Spectrally resolved fast transient brain states in electrophysiological data

**DOI:** 10.1016/j.neuroimage.2015.11.047

**Published:** 2016-02-01

**Authors:** Diego Vidaurre, Andrew J. Quinn, Adam P. Baker, David Dupret, Alvaro Tejero-Cantero, Mark W. Woolrich

**Affiliations:** a Oxford Centre for Human Brain Activity, Department of Psychiatry, University of Oxford, UK; b MRC Brain Network Dynamics Unit, Department of Pharmacology, University of Oxford, UK; c Computational Neuroscience, Department Biologie II Ludwig Maximilian, University Munich, Germany

**Keywords:** multivariate autoregressive model, MEG, Transient connectivity, Bayesian modelling, Spectral estimation, Multitaper, Coherence, Partial directed coherence, Sign ambiguity

## Abstract

The brain is capable of producing coordinated fast changing neural dynamics across multiple brain regions in order to adapt to rapidly changing environments. However, it is non-trivial to identify multiregion dynamics at fast sub-second time-scales in electrophysiological data. We propose a method that, with no knowledge of any task timings, can simultaneously identify and describe fast transient multiregion dynamics in terms of their temporal, spectral and spatial properties. The approach models brain activity using a discrete set of sequential states, with each state distinguished by its own multiregion spectral properties. This can identify potentially very short-lived visits to a brain state, at the same time as inferring the state's properties, by pooling over many repeated visits to that state. We show how this can be used to compute state-specific measures such as power spectra and coherence. We demonstrate that this can be used to identify short-lived transient brain states with distinct power and functional connectivity (e.g., coherence) properties in an MEG data set collected during a volitional motor task.

## Highlights

• We identify short-lived recurrent transient brain states of distinct multi-region spectral properties
• The method can be used to find time periods of quasi-stationary power spectra and phase locking
• The method can be used to produce denoised, task-locked time-frequency maps of power and coherence
• The approach is broadly applicable to any electrophysiological data in rest or task

## Introduction

The brain is able to coordinate neural oscillations across multiple brain areas in both rest and task (Buzsaki and Draguhn, 2004), (Mantini et al., 2007), (Fries, 2005), (Schnitzler and Gross, 2005). However, the manner in which these neural interactions arise in the brain is not fully understood. Typically, in electrophysiological data these oscillatory interactions are characterised using their multiregion spectral properties, e.g., the power content or the extent of phase locking (e.g., coherence) over different cortical regions (Lachaux et al., 1999). However, since the brain must be able to rapidly reorganise neural oscillations in response to the environment, there is a need to be able to identify how these multiregion spectral properties vary over time at potentially very fast (sub-second) time-scales.

Many existing methods for investigating time-varying patterns of spectral properties or functional connectivity use sliding time windows (Wendling et al., 2009), (Allen et al., 2014). Sliding window approaches pre-specify the temporal resolution of the changing patterns, and make inefficient use of the data when the same patterns occur recurrently at other points of time. The exception to this is when data can be pooled over epochs of a repeated task; however, this necessitates an assumption of stationarity over trials. These approaches also require a choice of the width of the time-window. Short windows can lead to noisy estimations, whereas long ones can miss the quickest changes.

In this paper, we provide a unified framework for characterising oscillatory dynamics in terms of their time-varying spatial and multiregion spectral properties without the knowledge of any task timings. The primary contribution of the method is that it operates simultaneously on the frequency, time and space dimensions, thus allowing for a unique description of transient spectral properties including power spectra and connectivity measures such as coherence. Importantly, it can identify when multiregion spectral patterns repeat at different points in time, and thereby pool over them to provide a better estimation of those patterns.

Although it is broadly applicable to any electrophysiological data modality, we focus here on magnetoencephalography (MEG), of particular interest for research on human connectivity for its fine-grain temporal resolution, wide-brain coverage and non-invasive nature. To this end, we also devise a way to deal with the sign ambiguity inherent to source reconstruction in MEG, which can jeopardise multisession/subject analyses if left unaddressed.

The method combines two well-known models: the multivariate autoregressive (MAR) (Penny and Roberts, 2002) model and the Hidden Markov model (HMM) (Juang and Rabiner, 1985). The MAR model characterises the behaviour of time series by linear historical interactions between the observed time series from different brain regions. MARs are able to characterise the frequency structure of the data, and by making the model multivariate, are able to capture interactions (e.g., coherence) between multiple brain regions. The HMM is a mathematical formalism that describes a time series as a sequence of states, where each state has its own model of the observed data (i.e., the observation model). Here, the observation model we use corresponds to a MAR model, and, hence, each state is related to a different set of multiregion autoregression coefficients describing the neural oscillations. In what follows, we will refer to the HMM with MAR observation model as the HMM–MAR.

Although the spectral contents of the states can be obtained directly from the (parametric) MAR model, we propose a non-parametric method based on the multitaper (Thomson, 1982) to obtain the states' spectral information given the state time courses. The motivations of the non-parametric approach are threefold. Firstly, the multitaper is known to provide a reliable estimation, often superior to the parametric approaches. Secondly, the MAR order, which is not needed for the non-parametric estimation, strongly affects the estimation of the spectral information. Finally, MAR orders that produce sensible state discrimination for the HMM–MAR do not necessarily match the MAR orders that are optimum for spectral estimation. We will show below that, even when the state visits are short (around 100 ms or less), the proposed statewise multitaper can provide reliable estimations of the entire range of frequencies of interest, including the low frequencies.

We first show how the model works on synthetic data, for which the ground-truth spectra are known. We then use the proposed model to characterise the neural dynamics in the primary motor cortex (M1) during a self-paced button press MEG experiment. We demonstrate that the proposed approach is able to identify HMM states that are task dependent despite training the HMM with no knowledge of the task timings, and that it can produce sensible state-specific estimates of the power spectral density (PSD), coherence and partial directed coherence (PDC) (Sameshima and Baccala, 1999) that are significantly different over states.

## The method

**Fig. 1:**
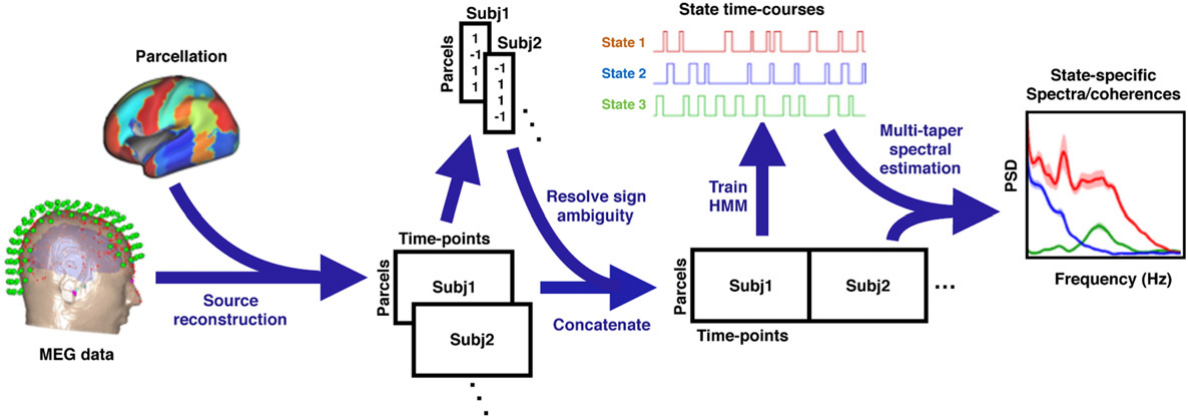
Workflow of the proposed method.

We now describe the HMM–MAR, its Bayesian hierarchy and some aspects of model selection and inference. We also provide details about the non-parametric spectral estimation, and about two issues that are central to source space MEG data analysis: sign ambiguity and signal leakage. Fig. 1 illustrates the proposed workflow schematically; each step is described below.

### Definition of the states and their Markov dynamics

In this section, we describe the observation model and the state transitions. As mentioned above, the observation model corresponds to a MAR model, and the state transitions follow the (first-order) Markovian assumption.

We first introduce some notation. Let ***y***_*t*_ ∈ ℝ^*N*^ be the multichannel source signal and *x*_*t*_ ∈ {1, …, *K*} the hidden state variable, with *t* = 1, …, *T*. Let A be the set of lags considered by the MAR model. We now present the MAR model leaving A unspecified, and will get into specifics about the choice of A in due course. Assuming Gaussian noise and centred data, our observation model is

(1) $$y{'}_{t}\left|x_{t}\right)=k\sim N\left(\sum_{l\in A}y{'}_{t-l}W_{l}^{\left(k\right)},\Sigma^{\left(k\right)}\right)\text{,}$$

where ***W***_*l*_^(*k*)^ are *N* × *N* dimensional matrices representing the *k*-th state autoregression coefficient matrices for lag *l* and the variance is given by some random noise distribution. We denote ***W***^(*k*)^ = [***W***_1_^(*k*)^; …; ***W***_*P*_^(*k*)^]. We shall also refer to the expectation of P(*x*_*t*_ = *k*|***Y***) as *γ*_*tk*_, and ***γ***_*t*_ = (*γ*_*t*1_, …, *γ*_*tK*_).

The noise covariance matrix **Σ**^(*k*)^ can be chosen to be diagonal or a full matrix. In the former case, we assume the zero-lag correlations to be zero. In the latter case, the noise is correlated across channels, which implies that the estimation of the autoregression coefficients has to be done for all channels at the same time (see Appendix B). Another decision to be made is whether we set the noise distribution to be equal for all states, so that **Σ** = **Σ**^(*k*)^, for all *k*.

**Fig. 2:**
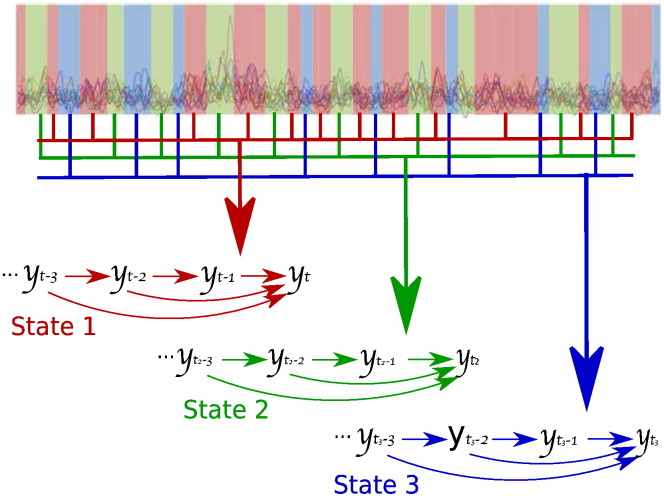
Graphical representation of the HMM–MAR. The time series (background) is partitioned into three states denoted by the blue, red and green slabs. Each state is characterised by a different set of dynamics, determined by the linear historical interactions between data points ***y***_*t*_ (small arrows).

For the hidden state variables, we use Markov dynamics, meaning that the probability *P*(*x*_*t*_ = *k*) is conditionally independent of the history of the state variable given *x*_*t* − 1_. Hence, we have

(2) $$P\left(x_{t}=k_{1}\left|x_{t-1}\right)=k_{2}\right)=\Theta_{k_{1}k_{2}},\;P\left(x_{1}=k\right)=\eta_{k}\text{,}$$

where $\Theta_{k_{1}k_{2}}$ and *η*_*k*_ are model paramerers that need to be inferred. The model is graphically represented in Fig. 2.

### Model complexity and model selection

In this section, we discuss the parametrisation of the MAR model and how to control its complexity. This is crucial, because, if the MAR models are too complex, the inference process (as a consequence of the Bayesian principle of parsimony) will tend to drop most of the states of the model by letting a few (or even one) dominant states to control the entire time series. Albeit good in terms of the tradeoff between predictability and parsimony, this hinders the discovery of quasi-stationary connectivity networks.

Firstly, driven by objective Bayesian principles, we use appropriate automatic relevance determination (ARD) priors on the autoregression coefficients. These ARD priors are Gaussian, and are imposed at two levels: for each lag (regularising on the time–frequency dimension) and for each pair of sources (regularising on the spatial dimension).

Secondly, we use incomplete MAR parametrisations. As indicated before, A represents the set of lags (see Eq. (1)). We now define *P* as the MAR maximum lag or order (i.e., all elements in A are lower than *P*). We have observed that the most common parametrisation, $A=\left\{1,2,\ldots ,P\right\}$, is not the most practical choice in this context. Due to the strong oscillatory components of MEG data and, for typical sampling rates, its high autocorrelation, it turns out that this configuration explains a high percentage of the data variance even for quite moderate values of *P*, and the explained variance keeps increasing asymptotically as we increase *P*. Instead, we use an exponential lapse *Q* and offset *P*_0_, such that $A=\left\{P_{0}+1,\left\lfloor P_{0}+Q\right\rfloor ,\left\lfloor P_{0}+Q^{2}\right\rfloor ,\ldots ,P\right\}$, where ⌊⌋ represents the floor operator. The exponential lapse allows us, with the same model complexity, to concentrate more statistical power on the lags with more autocovariance, i.e., on the lags that convey more information, without disregarding the lowest frequencies. On the other hand, given the high correlation between contiguous time points in MEG, choosing *P*_0_ > 0 greatly contributes to avoiding overfitting and the collapse of states. In all our experiments, *P*_0_ = 1 allows us to have twice the number of lags before saturating and produced a considerable improvement over *P*_0_ = 0.

We also consider the possibility of clamping certain connections to a certain fixed value so that they do not drive the state transitions. These connections can be fixed either to zero or to a (maximum likelihood) global value. This can be used to limit the complexity of the model, which can be necessary for computational purposes and in cases when the data are just too short, noisy or high-dimensional to permit a reliable estimation of the full model. Even more importantly, it is useful for investigating the transient dynamics of a particular set of connections. For example, we can force the HMM dynamics to be driven by just the PSD by setting to zero the cross-channel connections, or we can focus on coherence modulations by holding the diagonal elements of the autoregression coefficient matrices ***W***_*l*_^(*k*)^ to a fixed maximum likelihood value. We demonstrate below how this can be used to gain insight on real data. Appendix A gives some technical details about the formulation and implementation of this feature.

We also need to determine the number of HMM states, *K*. The strategy is to fix the maximum number, and then the Bayesian inference process can discard some of them if there is insufficient evidence to support their presence in the data. For the other parameters (*P* and *Q*), we could utilise the free energy (see Appendix B for derivations) or the cross-validated likelihood.

### The full Bayesian hierarchy

The observation model has been presented in Eq. (1), and the formulation of the state dynamics is illustrated by Eq. (2). We now proceed to detail the rest of the Bayesian hierarchy.

The noise of the signal is assumed to be Gaussian distributed with zero mean. We start by describing the covariance matrix of the noise, which can be assumed to be diagonal or a full matrix. When we assume a full covariance matrix, we model the precision matrix $\Omega^{\left(k\right)}=\Sigma^{{\left(k\right)}^{-1}}$ with a Wishart distribution,

(3) $$\Omega^{\left(k\right)}\sim W\left(\iota_{0},B_{0}\right)\text{.}$$

If we constrain the covariance matrix $\Sigma^{{\left(k\right)}^{-1}}$ to be diagonal, we have a Gamma distribution for each element of the diagonal,

(4) $$\omega_{ii}^{\left(k\right)}\sim G\left(\iota_{0},b_{0}\right)\text{.}$$

With interpretability in mind, we set a specific structure of group ARD priors on ***W***^(*k*)^. In particular, we expect each state to be characterised by a certain set of connections and certain frequency profile. Firstly, we use ARD precisions, *σ*_*ij*_^(*k*)^, to adaptively weight the presence of a specific connection between nodes (*i*, *j*) when in state *k*. The restriction *σ*_*ij*_^(*k*)^ = *σ*_*ji*_^(*k*)^ can be optionally imposed, depending on whether the focus is on direct or on undirected connections. Secondly, we use ARD precisions *α*_*l*_^(*k*)^ to adaptively weight the presence of interactions at a certain lag *l* for all nodes when in state *k*. The resulting Gaussian distribution for each element of the coefficient matrices is

(5) $$W_{l_{ij}}^{\left(k\right)}\sim N\left(0,\sigma_{ij}^{{\left(k\right)}^{-1}}\alpha_{l}^{{\left(k\right)}^{-1}}\right)\text{,}$$

with *σ*_*ij*_^(*k*)^ and *α*_*l*_^(*k*)^ being Gamma distributed

(6) $$\sigma_{ij}^{\left(k\right)}\sim G\left(\varphi_{0},c_{0}\right)\text{,}$$

(7) $$\alpha_{l}^{\left(k\right)}\sim G\left(\varsigma_{0},d_{0}\right)\text{.}$$

We denote ***σ***^(*k*)^ ∈ ℝ^*NN*^ = [*σ*_11_^(*k*)^, *σ*_1*N*_^(*k*)^; …; *σ*_*N*1_^(*k*)^, …, *σ*_*NN*_^(*k*)^] and ***α***^(*k*)^ = (*α*_1_^(*k*)^, …, *α*_*P*_^(*k*)^).

Note that there is an implicit definition of network under this structure of priors. More specifically, the formulation of ***σ***^(*k*)^ encourages each state to focus on a sub-set of connections, whereas ***α***^(*k*)^ controls the spectral dynamics for all nodes simultaneously, so that all the nodes within the network are encouraged to lie on the same frequencies.

Finally, the parameters that govern the state transitions are modelled as

(8) $$\Theta_{k\cdot }\sim Dir\left(\nu_{0}\right),\;\eta \sim Dir\left(\zeta_{0}\right)\text{,}$$

where **Θ**_*k* ⋅_ denotes the *k*-th row of **Θ**.

Eqs. (1), (2), (3), (4), (5), (6), (7), (8) jointly define the Bayesian hierarchy of the proposed HMM–MAR model. Appendix A discusses an extension of the model for holding some connections to a fixed value, as introduced in the previous section.

### Inference of the model parameters

The previous section completed the Bayesian hierarchy governing the model's parameters. Unfortunately, there is not a closed-form, analytical solution for the values of these parameters given the data. On these grounds, we use variational Bayes, which assumes additional factorisations in the space of parameters and needs all prior distributions to be conjugate (Bishop, 2006). Via an iterative algorithm acting on one group of parameters at a time, variational Bayes inference minimises the so-called free energy (Rezek and Roberts, 2005). This quantity is also useful for monitoring and model selection purposes.

The derivations are presented in Appendix B. Appendix C gives details about the computation of the free energy. Appendix D provides some insight about the initialisation of the model.

### Obtaining the HMM state-specific spectral properties

Once the training has converged and we have estimated the state time courses and the model parameters, there are two alternatives when it comes to finding out the multiregion spectral properties of each state: the parametric approach, which would use the MAR parameters learned during the training (see Appendix E for details), and the non-parametric approach, which instead takes advantage of the inferred HMM state time courses as temporal windows for estimating the state-specific spectral properties.

In this work, we choose to use a non-parametric approach. In particular, we propose a modification of the non-parametric multitaper method (Thomson, 1982) that works in the context of transient connectivity. The multitaper has been widely reported in the literature to present benefits in terms of accuracy and robustness (Mitra and Bokil, 2008). It reduces the frequency leakage inherent to finite-length sampling of the classical Fourier analysis by multiplying the time series by a function called taper, and then taking the Fourier transform. This is repeated with different orthogonal tapers, producing a number of power spectra estimations that are averaged later on. There are a number of possible choices for the taper function. We choose here the Slepian functions, a family of functions particularly appropriate for the multitaper. In our experiments, *R* = 7 tapers and a time–bandwidth product of 4 provides an adequate frequency resolution (0.4 Hz). We refer the reader to (Mitra and Bokil, 2008) for the impact of the choice of these parameters and other technical considerations.

We now describe how to use the state time courses to get state-specific spectral descriptions. The standard multitaper PSD estimate for the entire time series would be given by |**S**(*f*)|^2^, where

$$S\left(f\right)=\frac{1}{\sqrt{R}}\sum_{r=1}^{R}\sum_{t=1}^{T}\delta_{t}^{\left(r\right)}y_{t}e^{-2\pi i\mathrm{ft}}\text{,}$$

with *δ*_*t*_^(*r*)^ being the value of the *r*-th taper at time point *t*.

Our estimation for state *k*, that we denominate *statewise multitaper*, would instead be

$$S^{\left(k\right)}\left(f\right)=\frac{1}{\sqrt{R}}\sum_{r=1}^{R}\sum_{t=1}^{T}\rho_{t}^{\left(k\right)}\delta_{t}^{\left(r\right)}y_{t}e^{-2\pi i\mathrm{ft}}\text{,}$$

with

$$\rho_{t}^{\left(k\right)}=\frac{\sqrt{\gamma_{t}^{\left(k\right)}}}{\sqrt{\sum_{t=1}^{T}\gamma_{t}^{\left(k\right)}/T}}$$

giving thus more weight to those points in the signal that are more represented by state *k*. The normalisation term in the definition of *ρ*_*t*_^(*k*)^ is set such that, for every *k*, $\sum_{t}\rho_{t}^{\left(k\right)}\rho_{t}^{\left(k\right)}=T$, thus preserving the total power for each signal.

Finally, we can compute coherence directly using ***S***(*f*) exactly as we would do in the parametric case (Mitra and Bokil, 2008). To get the PDC non-parametrically is however not as straightforward, given that the PDC is defined in terms of the autoregression coefficients (Sameshima and Baccala, 1999). Instead, we make use of the Wilson algorithm to factorise the PSD produced by the multitaper into a unique minimum-phase transfer function, from which the computation of PDC is direct (Wilson, 1972), (Jachan et al., 2009). This is described in Appendix F.

Note that, as a consequence of the convolution theorem, the power spectrum of the state courses can mix up with the statewise spectra of the data. We have however observed that this is not much of a problem at least in the MEG data used in this paper, because the spectrum of the state time courses typically has most of the power lying in different frequency regions than the frequencies of interest in the data. Since this could be a issue in other applications of the HMM–MAR, we describe an alternative approach in Appendix E that can be used when the frequency content of the state time courses is strong enough to affect the multitaper estimation in the frequencies of interest. Essentially, this corresponds to re-inferring the spectra parametrically using a final iteration of the HMM–MAR, but using a complete, standard HMM–MAR without any missing lags. Please see Appendix E for a description of this parametric approach for power spectra estimation.

### Sign ambiguity

Source-localised MEG recordings suffer from an undesirable property: the equivalent current dipole model used in many source reconstruction techniques (e.g., beamforming) is unable to distinguish the polarity of a dipolar source, given that a dipole with a particular orientation will generate the same magnetic field pattern at the sensor array as one with the same orientation but opposite polarity. As such, the signs of the estimated source–space time course are arbitrary across cortical locations and across different recording sessions or subjects. As a result, the covariance between a pair of time courses may be positive in certain sessions, and negative in other sessions. This means that data from different sessions subjects cannot be straightforwardly compared or averaged at the group level. Whereas this does not present a problem when we use the amplitude envelope of the signals (Baker et al., 2014), this is a potential issue for MAR models, where we want to work with raw time courses.

In this paper, we propose to adjust the sign of the brain area time courses based on the assumption that the partial correlation between each pair of brain areas (channels) has the same sign across trials. We choose to use partial correlation instead of simple correlation because this is a direct measure, i.e., there are no other channels interfering in the “sign relation” between every pair of channels. Under this assumption, the correct combination of signs will be the one maximising the absolute sum of the partial correlations across all pairs of channels and trials, as the magnitude of this sum is obviously highest when the signs agree between trials. To find such combination of signs is an integer programming problem and, as such, its exact solution is NP-hard.

Here, we propose a heuristic search procedure that works as follows: we first initialise the signs randomly, computing the (stationary) precision matrix for each trial; then, until convergence, we look for the sign flipping that leads to the biggest increase of the absolute sum of partial correlations. This simple procedure is repeated a number of times, using multiple random starts to allow us to cover the search space sufficiently. For a moderate number of channels, this procedure is computationally efficient as it only implies the inversion of *N* × *N* matrices.

### The signal leakage issue

Another well-known problem for connectivity estimation in source space MEG data is the spurious leakage between sources. This is again a consequence of the ill-posed nature of the source reconstruction inverse problem, which aims to estimate the source activity from the sensor measurements (Schoffelen and Gross, 2009). To get around this problem we can choose a connectivity measure that is insensitive to zero-lag interactions, e.g., the imaginary part of coherency (Nolte et al., 2004) or phase-lag index (Stam et al., 2007). Alternatively, we can orthogonalise the time courses prior to estimating envelope correlation (Brookes et al., 2011), (Hipp et al., 2012), (Colclough et al., 2015). However, such approaches can be overconservative, as they remove genuine zero-lag correlation and, indirectly, also lagged correlations.

In the context of the HMM–MAR, we can take a different approach. Importantly, spatial leakage is induced by stationary correlations between source reconstruction weights. By contrast, the HMM–MAR is identifying states by virtue of how the time series change over time, and hence the HMM state identification cannot be influenced by the temporally stationary spatial leakage. However, since in the proposed pipeline (Fig. 1) we re-estimate the within-state spectral properties using the multitaper, the resulting state-specific functional connectivity measures (e.g., coherence) may be contaminated by spatial leakage. On these grounds, we only test for significance in spectral features (e.g., in Fig. 6) by looking for significant differences from the global time-averaged spectral features, thus subtracting out any spatial leakage effects.

## Simulations

We now present the performance of the approach on a relatively realistic synthetic dataset. This example will allow us to show that we can recover the slow frequency content using the proposed statewise multitaper even when the average duration of the states is shorter than the slowest wavelengths of interest.

We have used *N* = 2 channels and *K* = 3 states, each of which have a different MAR observation model with *P* = 35 uniformly-spaced lags, i.e., $A=\left\{1,2,\ldots ,35\right\}$. No exponential lags were considered in this example for simplicity. Diagonal (within channel) MAR coefficients were generated to produce coloured or 1/*f*^*α*^ noise using an iterative formula (Kasdin, 1995, Eq. 116). The three states were generated using *α*= .9, .7 and .5 respectively. Discrete oscillations are added to each channel by manipulating the roots of that channel's generating autoregressive parameters. Oscillations of 6, 12 and 18 Hz were added to, respectively, states one, two and three. Directional interactions on the off-diagonal of the MAR model were added to states by computing the dot product of the parameter matrix *W*^(*k*)^ with a *N* × *N* mixing matrix *A*. Whilst *A* is identity for state one (indicating no directional interactions), it contains a non-zero off diagonal term for states two and three,

$$A=\left\{\begin{array}{cc} \left[\begin{array}{ll} 1 & 0\\ 0 & 1 \end{array}\right] & \mathrm{if}\;k=1,\\ \left[\begin{array}{ll} 1 & 0\\ .5 & 1 \end{array}\right] & \mathrm{if}\;k=2,\\ \left[\begin{array}{ll} 1 & .5\\ 0 & 1 \end{array}\right] & \mathrm{if}\;k=3\text{,} \end{array}\right)$$

creating opposite directional interactions between the two nodes in each state. The spectral radius of the generated *W*^(*k*)^ was inspected to ensure that the generating MAR model is stationary.

We have sampled 100 trials of 4 s with a sampling frequency of 200 Hz, for a total of *T* = 100 × 4 × 200 = 80000 time points. Noise is white and independent for each channel. State lifetimes were drawn from a Gamma distribution with shape and duration parameters matched to the models fitted to the real data (Section Neural dynamics investigations in the primary motor cortex) shape = 1.48 and rate = 0.03. This ensures that each state is active for a length of time similar to what is typically observed. All three states occur with equal probability for the first and last 1500 ms of each epoch, whilst state 2 becomes more probable between 1500 and 2000 ms and state 3 becomes more probable between 2000 and 2500 ms, mimicking a ficticious event after 2000 ms of the start of each epoch. Once the state time courses are epoched around the event, these changes in probability will be expressed as an evoked change in the relative occupancy of each state around the centre of the epoch.

**Fig. 3:**
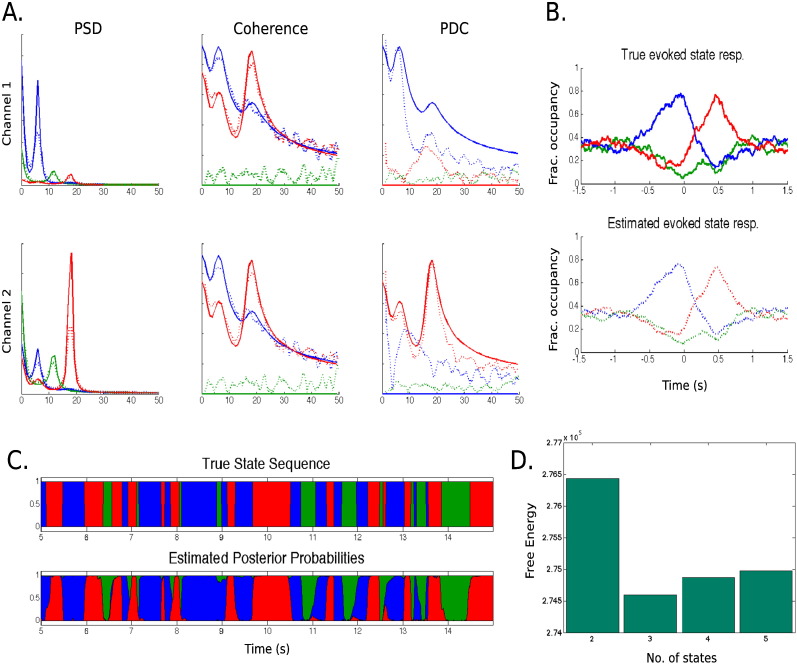
Results for the simulated data scenario. A. Ground truth frequency information (solid) alongside the multitaper estimation using the HMM–MAR inferred state time courses (dotted); note that the true red and green PDCs for channel 1 and the true blue and green PDCs for channel 2 are exactly zero. B. True (top) and estimated (bottom) average state responsibilities, locked to the event (*t* = 0). C. True (top) and estimated (bottom) state time courses for some portion of the data. D. Free energy for *K* = 2, 3, 4, 5.

Fig. 3A illustrates the estimated frequency information, including the PSD within each channel and the coherence and PDC between the channels. These frequency metrics are estimated from both the generating *W*^(*k*)^ matrix and the statewise multitaper estimation using the estimated state time series. By comparing these measures we can evaluate the extent to which the HMM–MAR inference and the statewise multitaper impact the spectral estimation. Qualitatively, the frequency estimations capture all the peaks in the simulated data with reasonable accuracy, though the PDC is noisier than the PSD or coherence estimations. Importantly, despite the relatively short state lifetimes, the estimation of the spectral metrics is good for both the lowest frequency peaks and the 1/*f*^*α*^ shape of the spectrum at very low frequencies.

Fig. 3B shows that the estimated fractional occupancy matches the true fractional occupancy quite accurately around the event. Fig. 3C shows in a segment of 10 s that, excepting for some of the faster transitions, the continuous inferred state time courses are also similar to the true state time courses.

Finally, Fig. 3D shows the free energy for *K* = 2, …, 5. As expected, the *K* = 3 model is the best model (lowest free energy) according to the free energy criterion.

The conclusions of this section are: (i) the statewise multitaper can detect frequencies that are on the order of the duration of the state visits, (ii) the approximate method for computing the PDC performs well, though is moderately noisier than coherence (see Appendix F for a description of the non-parametric PDC calculation).

## Neural dynamics investigations in the primary motor cortex

In voluntary movement performing, the M1 neural population undergoes a state of desynchronisation followed by a synchronisation period. These are usually referred to as event-related desynchronisation (ERD) and event-related synchronisation (ERS). In this section, we use the HMM–MAR and statewise multitaper to provide an alternative representation of the ERD/ERS in both time and frequency under a simple, volitional fingertapping movement.

### Data acquisition, preprocessing and HMM–MAR configuration

Ten right-handed volunteers (8 males and 2 females aged 25 ± 4 years) were asked to lie supine in the MEG system and execute a button press with the index finger of their non-dominant (left) hand. We selected eight of them on data quality grounds, rejecting two that, due to poor signal-to-noise ratio, did not show visible differences in activity in the button press. Subjects were instructed to repeat button presses infrequently (approximately once every 30 s) for a total of 1200 s, and not to count in the period between presses. Button presses were recorded using a keypad. The MEG data were acquired using a 275 channel CTF whole-head system (MISL, Conquitlam, Canada) at a sampling rate of 600 Hz with a 150 Hz low pass anti-aliasing filter. Synthetic third order gradiometer correction was applied to reduce external interference. The data were converted to SPM8 and downsampled to 200 Hz. Each recording was visually inspected to identify channels and/or periods of data containing obvious artefacts or with abnormally high variance, which were discarded. Independent component analysis (ICA) was used to remove components related to eye-blink and cardiac artefacts. Following artefact rejection the data were band-pass filtered between the 1 Hz and 48 Hz. The pre-processed data were projected onto a regular 8-mm grid spanning the entire brain using a scalar LCMV beamformer implemented in SPM8 (Van Veen et al., 1997), (Woolrich et al., 2011).

For each subject region of interest (ROI) within the left and right motor cortices were identified by localising the activity associated with the beta rebound. This was achieved by averaging the amplitude envelope of the data at each voxel in the 13–30 Hz band (computed via the Hilbert transform) within a time window 1 to 3 s after each button press. These time-averaged amplitude measures were baseline corrected by subtracting the average amplitude envelope within the time window 10 to 5 s before each button press. Finally, the baseline-corrected amplitude measures were averaged across all button press events, yielding a scalar value at each voxel. Subject specific regions of interest were defined as the maximum value of this statistic within the left and right hemispheres. Once these ROIs had been localised, time courses for the right and left hemisphere ROIs were obtained, again via the LCMV beamformer, within the wider 1–48 Hz band for use in the subsequent HMM–MAR analysis.

The HMM–MAR model was inferred using *K* = 3 states and maximal order *P* = 200, which corresponds to 1 cycle of the lowest data frequency (1 Hz). We used the free energy to choose the exponential lapse *Q*, resulting in $\left|A\right|=12$ autoregressive lags. We chose *P*_0_ to be 1, and we set **Σ**^(*k*)^ to be a diagonal matrix (and state-dependent). The results were relatively robust to reasonable variations of the chosen values, e.g., $\left|A\right|$ in between 10 and 16. For the reasons discussed in Section Model complexity and model selection, *P*_0_ = 1 introduces a considerable improvement over *P*_0_ = 0, with higher values of *P*_0_ regarded as unnecessary. The number of states, *K*, can also be chosen using the free energy. In this case, there were not any big differences in the free energy between *K* = 3 and moderately higher values of *K*. We hence chose *K* = 3 for ease of interpretation.

### Task-dependent state occurrence

We first consider the state time courses inferred by the HMM. These describe which state best represents the data at each point in time. Importantly, the HMM–MAR inference was performed in all cases with no knowledge of the task timings, i.e., in a completely unsupervised way. We can then epoch and average the state time courses, time-locked to the button-press. The resulting “fractional occupancy” reveals the proportion of trials for which the HMM–MAR was in a particular state, and we can examine these to see if the occurrence of states depends upon the task.

**Fig. 4:**
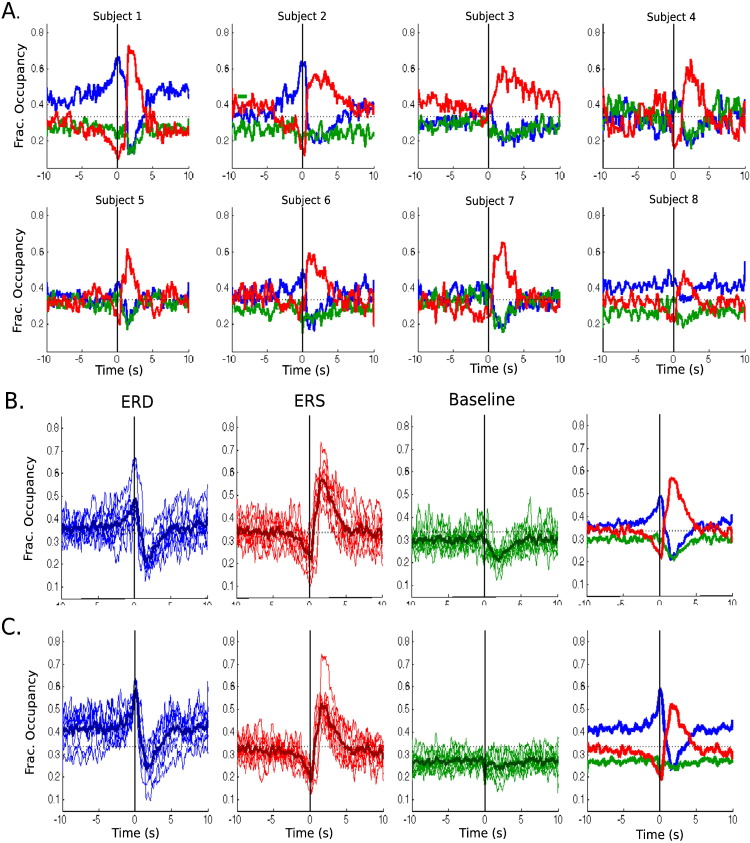
Fractional occupancy of the HMM–MAR states, labelled as ERD (blue), ERS (red) and baseline (green) according to their spectral properties and time of occurrence. A. Subject-by-subject individual runs, displayed by subject. B. Subject-by-subject individual runs, displayed by state. C. Group run (all subjects altogether), displayed by state. In B and C, the thick lines represent the mean across subjects. The rightmost panels of B and C depict just these means. Examples of raw state time courses, along with state life-time statistics, are shown in Fig. 8B, C.

Fig. 4A shows the result of this analysis with the HMM–MAR run individually on each subject. We denote the blue state as the ERD, the red state as the ERS (for reasons that become clear when we look at the spectral properties in the next section), and the green state can be considered “task irrelevant” baseline. These plots show that the HMM–MAR can produce states that are task dependent, despite the HMM being inferred with no knowledge of the task timings. Fig. 4A also reveals that there is a fair amount of subject-to-subject variability in the fractional occupancy time courses. For some subjects, for example, the ERD state is barely perceptible. The ERS state, although prominent in all cases, has a different shape for each subject. For the sake of comparison with the group results, Fig. 4B has the same information, but is organised in a different manner: the first three diagrams show, separated by states, the fractional occupancy for all subjects altogether (mean being represented by a thicker line), and the fourth diagram displays the mean state fractional occupancy averaged across subjects.

Fig. 4C shows the group result, for which the HMM–MAR model was estimated using the eight subjects simultaneously. The fractional occupancy here represents the proportion of trials spent in a particular state over all button presses and subjects. The results are not dramatically distinct from the individual runs, but some differences are still apparent, the most relevant being that the ERD state is more sharply activated around the button press and less active when we are far from the event.

### HMM–MAR regularised T–F analysis

**Fig. 5:**
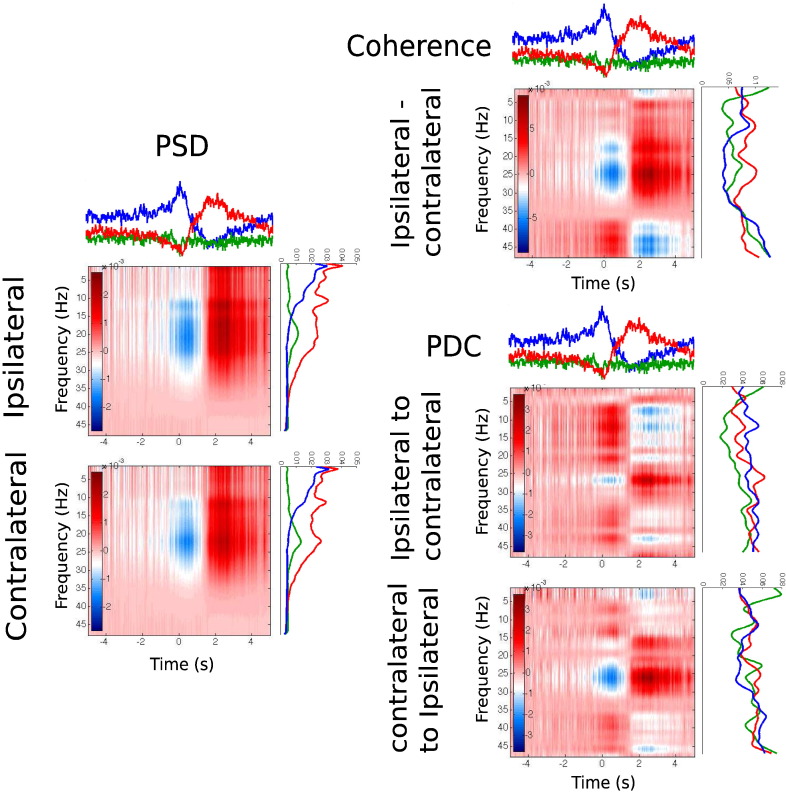
Schematic representation of the construction of time–frequency plots for PSD, coherence and PDC given the state time courses (on top of each panel) and the state frequency information (on the right of each panel).

Recall that, for each state, we have both the state time course and the state-specific spectral information obtained from the multitaper. Here we show how these two pieces of information can be pooled to construct HMM–MAR regularised time–frequency (T–F) representations. Fig. 5 shows this schematically: for each measure (PSD, coherence or PDC), a T–F plot is constructed as the sum over states of the outer product between each state time course (on top) and its corresponding measure values (on the right). For example, the PSD value for time point *t* and frequency bin *f* would be computed as ∑_*k* = 1_^*K*^PSD_*f*_^(*k*)^*γ*_*tk*_. All the PSD, coherence and PDC T–F plots were corrected to baseline, subtracting, for each frequency bin and subject, the mean value in between 5 s and 10s before the button press. Note that the statewise PSD/Coherence/PDC curves in the rotated axes are not baseline corrected, as correction is only performed within the corresponding T–F plot. Therefore, the resulting T–F plots can be thought of as T–F analyses regularised by the HMM–MAR inference.

**Fig. 6:**
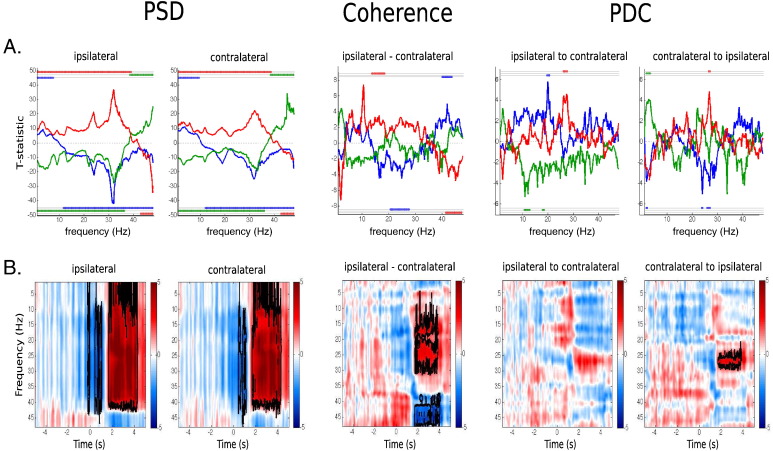
A. HMM–MAR states' frequency information (with standard error) for each state, with lines on top reflecting, for every pair of states, statistical significance (significance level of 0.05) of one state being higher than than the other; from top to bottom: ERD vs. ERS, ERS vs. baseline and ERD vs. baseline. B. Time–frequency representation of PSD, coherence and PDC, reconstructed from the state time courses and the state frequency raw values, where blue and red indicate, respectively, values that are lower and higher than baseline. 2D cluster based statistical significance (significance level of 0.05) is marked with black contours.

Fig. 6B shows statistical testing on these HMM–MAR regularised T–F representations, according to 2D cluster extent permutation testing (Maris and Oostenveld, 2007), where we used a cluster threshold of 3 and a significance level of 0.05. Areas of statistical significance (either significantly higher or lower than baseline) are marked with black contours. With regard to the PSD, we observe statistical significance in beta power that extends to the lowest frequencies and to low gamma, and a decrease in power shortly after the event. In terms of coherence, we can see statistically significant changes in beta (positive) and in gamma (negative) in between 2 s and 4 s after the event. PDC finds positive changes in beta that are statistical significant only for the contralateral-to-ipsilateral direction.

**Fig. 7:**
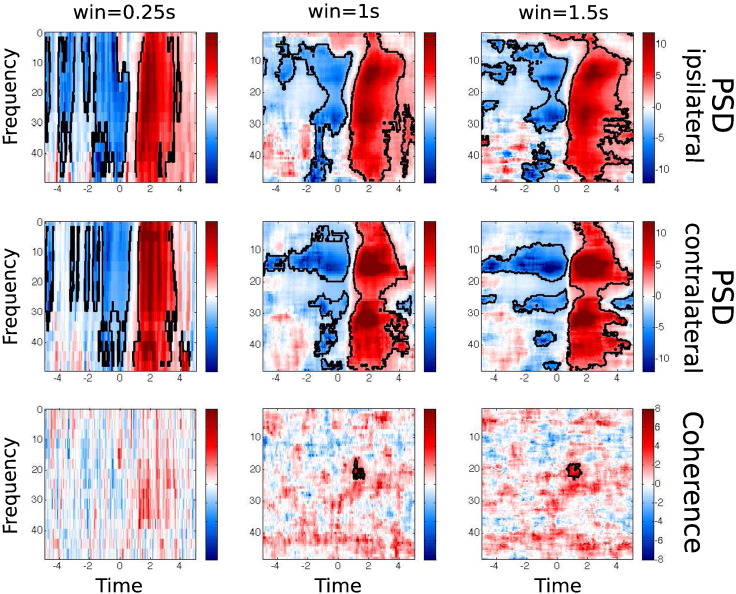
Time–frequency plots of PSD and coherence from the window multitaper, with sliding window lengths of 0.25 s, 1 s and 1.5 s. 2D cluster based statistical significance (significance level of 0.05) is marked with black contours.

For the sake of comparison, we also carried out a traditional T–F analysis using a standard sliding window-based multitaper. Given that this is using only a short period of time, these estimations are expected to be noisier than the ones produced by the HMM–MAR regularised method. Note also that PDC is missing. Because of the very high computational cost of the non-parametric PDC estimation (see Appendix F), its estimation over a sliding window is precluded in practice. Our method, by contrast, only needs *K* estimations of the PDC to produce a T–F PDC picture. Fig. 7 shows sliding window T–F PSD and coherence for different window sizes. For window sizes shorter than 1 s the estimations are quite noisy. Also, regardless the size of the window, T–F coherence turns out to be much noisier than the HMM–MAR regularised T–F coherence from Fig. 6B. Unlike the HMM–MAR regularised T–F estimation shown in Fig. 6B, standard T–F analysis shown in Fig. 7 fails to find differences in gamma coherence.

### State-specific spectral properties

As well as using the HMM–MAR to produce regularised T–F analyses, we can also examine the actual state-specific spectral information. This can reveal, for example, if there are state-specific differences in PSD or coherence.

Fig. 6A shows t-statistics comparing each state's spectral properties with the mean of the other two states. We assess statistical significance for each state, via two tests: one testing whether the value for the state is higher than the mean of the other two states (top lines), and other testing if it is lower (bottom lines). This is done according to cluster extent permutation testing (Maris and Oostenveld, 2007), with a cluster threshold of 3 and significance level of 0.05.

Overall, Fig. 6A reveals a number of statistically significant state-specific differences in PSD, coherence and PDC. In particular, the ERS state has a higher power across the entire alpha and beta range for both ipsilateral and contralateral M1, with no strong differences between hemispheres with regard to statistical significance. Both the ERS and the ERD states have significantly less power in low gamma (from around 42 Hz) than baseline. There are also state-specific differences in coherence. For example, the ERS state shows more coherence in beta (between 14 and 19 Hz), and less coherence in low gamma (around 40 Hz). The ERD state, conversely, has more coherence in low gamma (around 40 Hz), and less coherence in beta (20–28 Hz).

### Comparisons of different HMM observation models

In this section we aim to understand which particular aspects of the data are driving the state transitions by comparing three different versions of the HMM–MAR. We refer to HMM–Gaussian as the model with Gaussian observations on the power (Hilbert envelope) of the signals (Baker et al., 2014), HMM–AR as the model for which only the self-autoregression coefficients (the diagonal elements of ***W***_*l*_^(*k*)^) are allowed to vary across states, and HMM–crossMAR as the model where the self autoregression coefficients were set to remain fixed and the cross-channel coefficients were solely responsible for driving the state transitions. Whereas the HMM–AR is driven by the PSD, the HMM–crossMAR would be driven by functional connectivity (e.g., coherence).

**Fig. 8:**
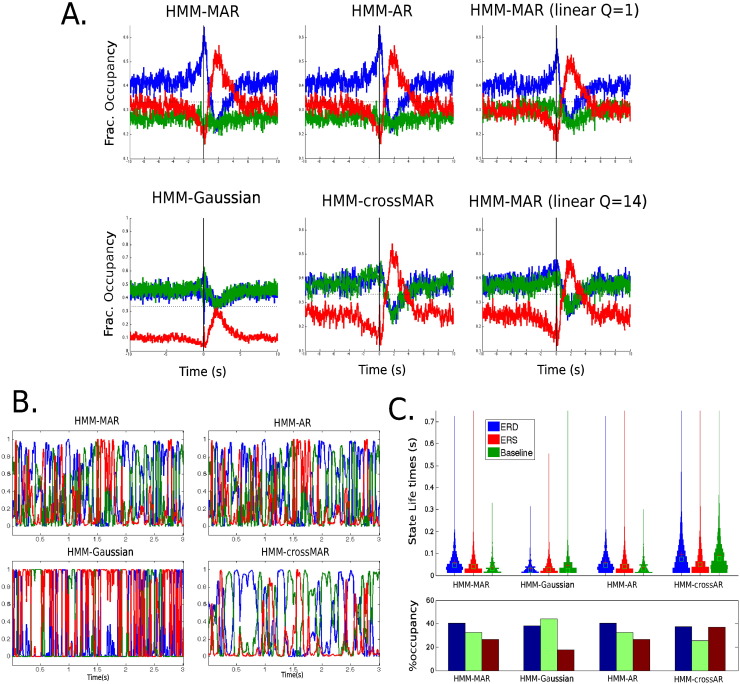
A. State fractional occupancy around the button press for different variations of the HMM–MAR: the HMM–Gaussian run on the Hilbert envelopes of the signal, an HMM–MAR where only the self-autoregression coefficients were allowed to vary, an HMM–crossMAR where only cross-channel coefficients were allowed to vary, and the HMM–MAR with uniformly-spaced lapses for *Q* = 1 and *Q* = 14. B. Examples of the state time courses for the HMM–Gaussian, HMM–MAR, HMM–AR and HMM–crossMAR. C. State life times and percentage of state occupancy for the HMM–Gaussian, HMM–MAR, HMM–AR and HMM–crossMAR.

Fig. 8A displays the state fractional occupancy around the button press for the different alternative parametrisations. Note that the HMM–Gaussian fails to recover the dynamics around the button press, although it can identify changes at the ERS time. Interestingly, the HMM–AR estimation is effectively equal to the full HMM–MAR estimation, suggesting that the autocorrelation (or PSD) has enough information by itself to drive the state transitions meaningfully. Notably, the HMM–crossMAR is also able to identify the ERS. This shows that changes in functional connectivity alone are sufficient to identify fast transient brain states that are task related. However, the HMM–crossMAR is unable to pick up the ERD satisfactorily.

We have also tested a uniformly-spaced lapse regime, instead of the exponential lapse used all through the rest of the experiments. As with the exponential lags, we use 12 lags. The HMM–MAR with a uniformly-spaced lapse fails to identify the ERD state (which gets mixed up with the baseline state) when we set it to cover the entire 1 Hz cycle (lapse of 14 time points). If we set the lapse to be 1, the identification of the lowest frequencies becomes harder, but (although less sharply) it still can differentiate the ERD/ERS.

Fig. 8B shows examples of the state time courses, and Fig. 8C presents their mean state life times and occupancies. Note that the state time courses are practically identical for the HMM–MAR and the HMM–AR. The state time courses are sharper for the HMM–Gaussian, in the sense that they take values mostly close to either zero or one, whereas they are smoother and fuzzier for the HMM–crossMAR. State life times are a bit longer for the HMM–MAR than for the HMM–Gaussian, and they are the longest for the HMM–crossMAR.

## Discussion

### Summary of the contributions and related work

In this paper, we have proposed an approach for characterising patterns of oscillatory activity that vary across time, frequency and cortical location. Generally speaking, the delimitation of a particular pattern of activity (or functional network) is not straightforward unless we fix one of these dimensions, by assuming temporal stationarity or by looking at the oscillatory power within a particular frequency band (Baker et al., 2014), (Brookes et al., 2011). The HMM–MAR overcomes this limitation by dealing with time, frequency and cortical location simultaneously. Once the HMM–MAR is estimated, we make use of a weighted version of the multitaper to estimate a range of multiregion spectral characteristics. This enables us to benefit from the advantages of both parametric and non-parametric approaches. We show how this can be used to create regularised time–frequency analyses of PSD, coherence and PDC; as well as to identify fast transient states with multiregion spectral properties that are task dependent.

The HMM–MAR is related to, and builds upon, other approaches. A Bayesian approach of the stationary MAR model has been introduced by Penny and Roberts (2002). Astolfi et al. (2008) have extended this to produce temporally non-stationary MAR models to obtain brain connectivity patterns that, instead of sequentially switching between a set of brain states, change smoothly over time. Baker et al. (2014) have previously used an HMM with a Gaussian observation model to discover whole-cortex resting state networks from MEG, obtaining networks that resemble those previously found with fMRI. MAR models have also previously been used as observation models for HMMs in the context of speech processing (Juang and Rabiner, 1985), for neural signal analysis (Cassidy and Brown, 2002), and in the signal processing literature (Fox et al., 2011).

### Findings on the M1 neural dynamics

We have shown that the HMM–MAR can reveal fast changing spectral information in a volitional motor task. Whilst the purpose of the inclusion of the motor task is to allow us to qualitatively demonstrate the new information that can be extracted using the proposed method, we can briefly consider some of the implications of the findings that may be worthy of further investigation. For example, the model can detect the ERD starting to build up around 2.5 s before the button press. Such an early onset may be a consequence of a selection bias (Schurger et al., 2012). With regard to the frequency information, it is generally accepted that alpha/mu and beta are the most significant rhythms in voluntary movement. Using the HMM–MAR, we show, consistent with some previous work (Cheyne et al., 2008), that higher frequencies (in the gamma band) might also be involved in the mechanisms of motor control. These results are obviously limited by the fact that the data we have used for our experiments only covers up to 48 Hz. Also, we show that there are changes at the level of coherence and directed coherence between the two hemispheres that were difficult, or not at all possible, to observe using traditional sliding window time–frequency analysis techniques.

At any rate, it is not the objective of the present paper to perform a thorough analysis of the neural dynamics of voluntary movement. However, we note that the HMM–MAR has the capability to provide insights into multiregion spectral patterns of response in task experiments in general. For example, considering that some studies have shown that the location of the beta ERS peak is different from the maximal alpha ERD (Pfurtscheller et al., 1996), (Salmelin et al., 1995), (Jurkiewicz et al., 2006), an interesting further line of research would be to assess the spatial source of the different rhythms. A more comprehensive selection of ROIs could provide further insights than the current use of just left/right motor cortex.

Note that the model is effectively estimated on a combination of task and rest, the latter corresponding to long periods of baseline between the short button press events. We observed that, although the ERD/ERS states are dominant in the surroundings of the button press, they are also occurring in the rest (baseline) periods of the experiment. This is consistent with previous work on MEG data that revealed fast switching between different brain states on 100 ms timescales within the resting state (Baker et al., 2014). Here, we are observing a similar phenomena, albeit limited in this case to the left/right motor cortex. The structure of this baseline state switching is worth investigating as it can be related to the hypothesis that the same states that are needed for task are also visited at rest (Kenet et al., 2003), (Smith et al., 2009).

### Limitations, future work and other applications

Since the HMM models the brain activity as switching between a limited set of discrete states, it is interesting to consider what will happen in a task with continuously varying levels of task or stimulation (e.g., in a visual task where a grating is presented with a variety of different contrast levels). One strong possibility is that the different contrast levels would straightforwardly be captured using differences in the fractional occupancy (i.e., the proportion of time that the brain visits a particular state). Indeed, this is what can be seen to happen when the brain switches from a baseline state into the task state. However, it is also possible that, in certain situations, the response of the system to different contrast levels could be better modelled by a modulation of a state's “strength” (e.g., the power in that state) whilst the fractional occupancy remains fixed. Exploring this alternative possibility would require a different HMM observation model, which allows for scaling (e.g., of the PSDs that each state's MAR represents) and that varies at a slower time-scale than the state-switching. For example, this could be achieved using a hierarchical model augmented to the current framework.

EEG/MEG data have been shown to exhibit scale-free behaviour (Van De Ville et al., 2010), (Gschwind et al., 2015), a feature that is common in the neural system at multiple levels, both anatomical and functional (Buzsaki and Mizuseki, 2014). Although the HMM paradigm is efficient at capturing short-term dependencies, it does not explicitly model long-term dependencies that are characteristic of scale-free systems because of the limitations of the first-order Markov assumption. However, despite this, it is still very possible that the inferred HMM state time course (as in EEG microstates) can still exhibit scale-free properties. This is an area for future investigation.

Another consideration with the proposed method is that, similarly to what happens with ICA, matching states between runs that have been done independently for different subjects can be non-trivial. In our experiments, this is not a problem because all our results (with the exception of Fig. 4) come from group estimations on data concatenated over subjects. In other cases, it might be preferable to have individual runs due, for example, to computational reasons. One can then use a prediction-based measure, where we give the same (random) inputs to all MAR models and then compare the predictions. Provided this is done for a sufficiently high number of inputs, the prediction-based approach is a quite general and free of assumptions.

Finally, we recall that the proposed statewise multitaper estimation conveys information from both the data time series and the state time courses. Since our estimation derives from a point-wise multiplication in the time-domain, which would relate to a convolution between the spectrum of the time-series and the spectrum of the state time courses, the estimated statewise multitaper spectra might be distorted if the frequency spectrum of the state time courses contains significant structure. This was not the case in neither our synthetic simulations nor our study of the primary motor cortex, but it could happen in other scenarios. By all means, it is recommendable to inspect the spectra of the state time courses and, if this happens to be a problem, to use the parametric estimation, detailed in Appendix E.

Considering that simpler HMM-based models have already been proven useful for unveiling resting-state networks in MEG (Baker et al., 2014), a natural next step is to use the HMM–MAR to study whole brain resting-state dynamics. An obvious problem here is the dimension of the data: if we aim to analyse a big number of neural sources simultaneously, the number of parameters escalates rapidly and hinders the estimation. To overcome this limitation, we can fix some of the MAR parameters, as discussed above. For example, one possibility is to let only the self-connections drive the state transitions, reducing the number of parameters from *K* × *PN*^2^ to *K* × *PN*. In this case, each state is reduced to a collection of unrelated autoregressive processes. Alternatively, we might consider a dimensionality reduction more appropriate to transient dynamics than PCA, possibly by including the estimation of the PCA weights into the HMM–MAR inference process. Another different direction is to use these ideas to model fMRI data, for which, ideally, we would incorporate the hemodynamic response function into the model.

A further interesting potential application of the HMM–MAR is about prediction. The method could be used in the situation where we have pre-specified the value of the hidden state for segments of the time series, so that this remained fixed all through the learning process. In a typical application we would have labelled and unlabeled trials, the objective being to assign labels to the unlabelled ones. This can considered a case of semi-supervised learning and is of potential interest, for instance, in the field of brain–computer interfaces (Wolpaw et al., 2002). To mention one possible example, we could use the proposed technique to provide online prediction of epileptic fits based on the information of previous seizures. Building on previous knowledge, we could specify the moments previous to the seizure as a given state so that, at any moment, we would have an online estimation of the probability of being at risk.

## Figures and Tables

**Fig. 9:**
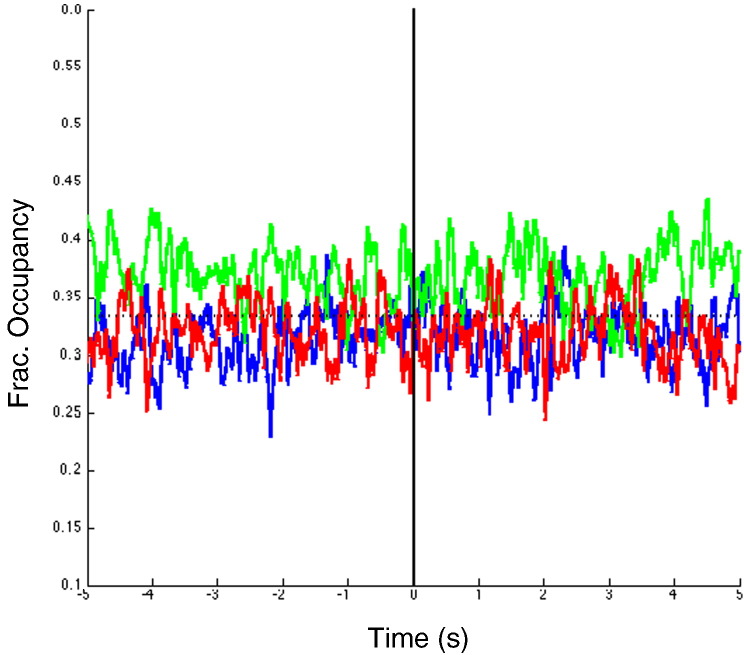
Average state time courses around the button press event, estimated from an HMM–MAR run on a data set that was sampled from a MAR model. This MAR model is such that can explain up to 99.99% of the original data variance.

## Appendix A Holding connections to a fixed value

As discussed above, we can hold certain connections so that they do not drive the state transitions. These connections can be fixed either to zero or to a (maximum likelihood) global value. In this case, the model from Eq. (1) becomes

$$y{'}_{t}\left|x_{t}\right)=k\sim N\left(\sum_{l\in A}y{'}_{t-l}\left(G^{\left(0\right)}\cdot W_{l}^{\left(0\right)}\right)+\sum_{l\in A}y{'}_{t-l}\left(G\cdot W_{l}^{\left(k\right)}\right),\Sigma^{\left(k\right)}\right)\text{,}$$

where ∙ denotes element-wise matrix product and ***W***_*l*_^(0)^ is the *l*-lag global autoregressive coefficient matrix obtained, for instance, using maximum likelihood (least squares estimation). We let ***G*** be a *N* × *N* indicator matrix whose elements are 1 if the corresponding connection is allowed to vary across states and 0 otherwise. Likewise, ***G***^(0)^ is another *N* × *N* matrix indicating which connections are modelled globally.

This formulation allows us to fix some connections to maximum likelihood by setting *G*_*ij*_ = 0, *G*_*ij*_^(0)^ = 1 and to zero by setting *G*_*ij*_ = 0, *G*_*ij*_^(0)^ = 0. Although we do not explore this alternative in this paper, it also permits to discount the global trends before modelling transient connectivity by setting *G*_*ij*_ = 1, *G*_*ij*_^(0)^ = 1.

To proceed with the inference, we first obtain the estimation ***Ŵ***_*l*_^(0)^ using least squares and then we define new variables

$$r{'}_{t}=y{'}_{t}-\sum_{l\in A}y{'}_{t-l}\left(G^{\left(0\right)}\cdot {\hat{W}}_{l}^{\left(0\right)}\right)\text{,}$$

so that, all through the inference procedure, instead of the model in Eq. (1), we use the following state model

$$r{'}_{t}\left|x_{t}\right)=k\sim N\left(\sum_{l\in A}y{'}_{t-l}\left(G\cdot W_{l}^{\left(k\right)}\right),\;\Sigma^{\left(k\right)}\right)\text{.}$$

In Appendix B, we describe the variational Bayes inference equations. For simplicity, inference is specified for the standard case ***G*** = 1, ***G***^(0)^ = 0, where there is no need to define additional variables ***r***'_*t*_. The extension to cover this generalisation of the model is straightforward.

## Appendix B Variational Bayes inference

We use the variational approach for inferring the parameters of the model (Bishop, 2006), alternating a (variational) E-step and M-step. The variational E-step deals with the estimation of the hidden states probabilities and the variational M-step estimates the model parameters. Convergence is guaranteed for this kind of algorithms and can be monitored by means of the free energy of the model. The computation of the free energy is discussed in Appendix C.

We next show the variational updating equations for the special case when there is one single batch or trial. The following equations can easily be adapted to the general case, when more than one time series are fed to the algorithm.

We denote the entire source signal as **Y** ∈ ℝ^*T* × *N*^ and the vector of states as ***x***. Also, we define ***Y***^+^ = [***y***'_*P* + 1_; …; ***y***'_*T*_] ∈ ℝ^*T* − *P* × *N*^, ***Y***_*i*_^−^ = [***y*** ' _*P* − *i* + 1_; …; ***y*** ' _*T* − *i*_] ∈ ℝ^*T* − *P* × *N*^ and ***Y***^−^ = [***Y***_1_^−^ … ***Y***_*P*_^−^] ∈ ℝ^*T* − *P* × *NP*^. We also denote **Γ**^(*k*)^ = diag(***γ***_*k*_).

We start outlining the M-step. For the observation model, we approximate the posterior distribution of the parameters P(***W***, **Ω**, ***σ***, ***α***|***s***, ***Y***) by a variational distribution with the following factorisation

(9) $$F\left(W,\Omega ,\sigma ,\alpha \right)=F\left(W\right)F\left(\Omega ,\sigma ,\alpha \right)\text{.}$$

Without further assumptions, a factorisation between **Ω**, ***σ*** and ***α*** follows naturally.

*F*(**Ω**^(*k*)^) corresponds to a Wishart probability distribution

$$F\left(\Omega^{\left(k\right)}\right)=W\left(B^{\left(k\right)},\iota^{\left(k\right)}\right)\text{,}$$

with *ι*^(*k*)^ = *ι*_0_ + ∑_*t* = *P* + 1_^*T*^*γ*_*tk*_ and ***B***^(*k*)^ = (***E***^(*k*) '^**Γ**^(*k*)^***E***^(*k*)^ + ***B***_0_)^− 1^. We define the residuals $E^{\left(k\right)}=Y^{+}-Y^{-}\mu_{W^{\left(k\right)}}$, and $\mu_{W^{\left(k\right)}}$ is the expectation of the autoregression coefficients. We denote the expectation of **Ω**^(*k*)^ as ${\bar{\Omega }}^{\left(k\right)}=\iota^{\left(k\right)}B^{\left(k\right)}$.

We define ${\tilde{w}}^{\left(k\right)}$ as the concatenation of the rows of ***W***^(*k*)^ and ${\tilde{w}}^{{*}^{\left(k\right)}}$ as the concatenation of the rows of the *NP* × *N* least-squares solution

$${\left(Y^{-'}\Gamma^{\left(k\right)}Y^{-}\right)}^{-1}Y^{-'}\Gamma^{\left(k\right)}Y^{+}\text{.}$$

Let **Φ**^(*k*)^ be a diagonal matrix defined as follows: we build vectors *ϕ*_*l*_^(*k*)^ as the concatenations of the columns of the square matrices ${\bar{\alpha }}_{l}^{\left(k\right)}{\bar{\sigma }}^{\left(k\right)}$; we then construct **Φ**^(*k*)^ as the diagonal matrix whose diagonal elements are the concatenation of vectors *ϕ*_*l*_^(*k*)^. After some algebraic manipulations, we can identify $F\left({\bar{w}}^{\left(k\right)}\right)$ as *N*^2^*P*-dimensional Gaussian distribution

(10) $$F\left({\bar{w}}^{\left(k\right)}\right)=N\left(\mu_{{\tilde{w}}^{\left(k\right)}},S_{{\tilde{w}}^{\left(k\right)}}\right)$$

with $S_{{\tilde{w}}^{\left(k\right)}}={\left(\Phi^{\left(k\right)}+Y^{-'}\Gamma^{\left(k\right)}Y^{-}⊗{\bar{\Omega }}^{\left(k\right)}\right)}^{-1}$, $\mu_{{\tilde{w}}^{\left(k\right)}}=S_{{\tilde{w}}^{\left(k\right)}}\left(Y^{-'}Y^{-}⊗{\bar{\Omega }}^{\left(k\right)}\right){\tilde{w}}^{*\left(k\right)}$, where ⊗ represents the Kronecker product. From this expression, we can easily reconstruct $F\left(W^{\left(k\right)}\right)=N\left(W^{\left(k\right)};\mu_{W^{\left(k\right)}},S_{W^{\left(k\right)}}\right)$.

For *σ*_*ij*_^(*k*)^, and assuming symmetry *σ*_*ij*_^(*k*)^ = *σ*_*ji*_^(*k*)^, we have a Gamma distribution

(11) $$F\left(\sigma_{ij}^{\left(k\right)}\right)=G\left(\varphi_{ij}^{\left(k\right)},c_{ij}^{\left(k\right)}\right)$$

with rate ${\tilde{c}}_{ij}^{\left(k\right)}=\frac{1}{2}\sum_{l=1}^{P}{\bar{\alpha }}_{l}^{\left(k\right)}\left(\mu_{W_{l_{ij}}^{\left(k\right)}}^{2}+S_{W_{l_{ij}}^{\left(k\right)}}+\mu_{W_{l_{ji}}^{\left(k\right)}}^{2}+S_{W_{l_{ji}}^{\left(k\right)}}\right)+c_{0}$ and shape *φ*_*ij*_^(*k*)^ = *P* if *i* ≠ *j* and $\varphi_{ij}^{\left(k\right)}=\frac{P}{2}$ otherwise. The derivation is similar for *σ*_*ij*_^(*k*)^ ≠ *σ*_*ji*_^(*k*)^.

For *α*_*l*_^(*k*)^, we have

(12) $$F\left(\alpha_{l}^{\left(k\right)}\right)=G\left(\varsigma_{l}^{\left(k\right)},d_{l}^{\left(k\right)}\right)$$

with rate ${\tilde{d}}_{n}^{\left(k\right)}=\frac{1}{2}\sum_{i,j=1}^{N}{\bar{\sigma }}_{ij}^{\left(k\right)}\left(\mu_{W_{l_{ij}}^{\left(k\right)}}^{2}+S_{W_{l_{ij}}^{\left(k\right)}}\right)+d_{0}$ and shape $\varsigma_{ij}^{\left(k\right)}=\frac{N^{2}}{2}$.

We are now in a position to specify the marginal expectation of ***y***'_*t*_ as

(13) $$E\left[y{'}_{t}\right]=\sum_{k=1}^{K}\sum_{l\in A}\gamma_{tk}y{'}_{t-l}W_{l}^{\left(k\right)}\text{.}$$

Note that, because the state time courses are probabilistic, the expectation is effectively a mixture of the *K* states, where the weights are given by *γ*_*tk*_.

The estimation of the HMM hidden state sequence probabilities conforms the variational E-step of the algorithm. The regular forward–backward recursions (Rabiner, 1989), adapted for the variational approach, can be used for this matter. We need the conditional likelihoods

$$\begin{array}{c} \log L_{t}^{\left(k\right)}=\mathrm{logP}\left(y_{t}\left|x_{t}\right)=k,W^{\left(k\right)}\Omega^{\left(k\right)}\right)\\ =-0.5N\log\left(2\pi \right)-0.5\log\left|B^{\left(k\right)}\right|+0.5\sum_{i=1}^{N}\psi \left(0.5\iota^{\left(k\right)}-0.5i+0.5\right)-0.5N\log2+\\ \;-0.5\;\mathrm{diag}\left(E\left[e_{t}^{\left(k\right)'}\left(\iota^{\left(k\right)}B^{\left(k\right)}\right)e_{t}^{\left(k\right)}\right]\right) \end{array}$$

where *ψ*(⋅) is the digamma function.

The forward–backward equations make use of the message variables

$$\begin{array}{l} \beta_{f}\left(x_{t}\right)=P\left(y_{1},\ldots ,y_{t},x_{t}\right),\\ \beta_{b}\left(x_{t}\right)=P\left(y_{t+1},\ldots ,y_{T},x_{t}\right)\text{.} \end{array}$$

The forward recursions can be calculated, for *t* = *P* + 1, …, *T*, as

$$\beta_{f}\left(x_{P+1}\right)=\bar{\eta }L_{P+1}^{\left(k\right)},\;\beta_{f}\left(x_{t}\right)=\left(\beta_{f}\left(x_{t-1}\right)\bar{\Theta }\right)\cdot L_{t}^{\left(k\right)}\text{,}$$

where ∙ denotes the elementwise product and $\bar{\Theta }=E\left[\Theta \right]$. For the backward recursions, for *t* = *T* − 1, …, *P* + 1, we have

$$\beta_{b}\left(x_{T}\right)=\frac{1}{{\left\|\beta_{f}\left(x_{T}\right)\right\|}_{1}}1_{K},\;\beta_{b}\left(x_{t}\right)=\frac{1}{{\left\|\beta_{f}\left(x_{t}\right)\right\|}_{1}}\left(\beta_{b}\left(x_{t+1}\right)\cdot L_{t+1}^{\left(k\right)}\right){\bar{\Theta }}^{\prime }$$

where 1*_K_* is a vector with all elements equal to 1 and |**v**|_1_ is the sum of the elements of the vector **v**.

So, at the E-step, we compute the forward messages, starting from *t* = *P* + 1, and, then, we compute the backward messages from *t* = *T* to *P* + 1, combining them to get

$$\gamma_{t}=\frac{\beta_{b}\left(x_{t}\right)\cdot \beta_{f}\left(x_{t}\right)}{{\left\|\beta_{b}\left(x_{t}\right)\cdot \beta_{f}\left(x_{t}\right)\right\|}_{1}}\text{.}$$

We also compute variables ***ξ***_*t*_ ∈ ℝ^*KK*^ such that

$$\xi_{t\;k_{1}\;k_{2}}=E\left[P\left(x_{t}=k_{1},x_{t+1}=k_{2}\left|Y\right)\right)\right]={\bar{\Theta }}_{k_{1}\;k_{2}}\;\beta_{f}\left(x_{t}=k_{1}\right)\beta_{b}\left(x_{t+1}=k_{2}\right)L_{t+1}^{\left(k_{2}\right)}\text{.}$$

The parameters of the variational Dirichlet distribution that governs the state dynamics, $F\left(\Theta_{k_{1}\cdot }\right)=Dir\left(\nu_{k_{1}\cdot }\right)$ and *F*(***η***) = *Dir*(***ζ***), are updated as

$$\nu_{k_{1}k_{2}}=\frac{\exp\left(\psi \left({\bar{\xi }}_{k_{1}k_{2}}\right)-\psi \left({\left\|\bar{\xi }\right\|}_{1}\right)\right)}{\sum_{k=1}^{K}\exp\psi \left(\left({\bar{\xi }}_{k_{1}k}\right)-\psi \left({\left\|\bar{\xi }\right\|}_{1}\right)\right)}\text{,}$$

where $\bar{\xi }=\nu_{0}+\sum_{t=P+1}^{T-1}\xi_{t}$, and

$$\zeta_{k_{1}}=\frac{\exp\left(\psi \left(\gamma_{P+1\;k_{1}}\right)-\psi \left({\left\|\gamma_{P+1}\right\|}_{1}\right)\right)}{\sum_{k=1}^{K}\exp\left(\psi \left(\gamma_{P+1\;k}\right)-\psi \left({\left\|\gamma_{P+1}\right\|}_{1}\right)\right)}\text{.}$$

## Appendix C Free energy computation

The free energy F is the cost function that variational inference aims to minimise. F is typically used to monitor the convergence of the algorithm and for model comparison and selection. It can be derived as the sum of the model average log-likelihood, the negative entropy and the Kullback–Leibler divergence,

Expressions for all these quantities can be found for example in Rezek and Roberts (2005), where the HMM with Gaussian observations (among others) is presented. Note that the HMM–MAR can be considered a generalisation of this simpler model. With regard to the computation of F, we need to consider that the predicted value is not just the mean of a Gaussian distribution but the autoregressive prediction given by Eq. (13). The total Kullback–Leibler divergence, as calculated in Rezek and Roberts (2005), needs also to include the Kullback–Leibler divergence between:

• The variational posterior distribution of **W**^(*k*)^ given in Eq. (10) and the prior distribution given by Eq. (5) (Gaussian).
• The variational posterior distribution of ***σ***_*ij*_^(*k*)^ given in Eq. (11) and the prior distribution given by Eq. (6) (Gamma).
• The variational posterior distribution of ***α***_*l*_^(*k*)^ given in Eq. (12) and the prior distribution given by Eq. (7) (Gamma).

The expressions of the Kullback–Leibler divergence for the Gaussian and Gamma distributions can also be found in Rezek and Roberts (2005).

## Appendix D State course initialisation

The algorithm described above needs an initial estimation of ***γ***_*k*_. We contemplate different choices for this. The first one (and the simplest) is a mixture of Gaussian model (Bishop, 2006), which can be itself initialised with a K-nearest neighbour algorithm. The second, that can be considered an extension of the former, is a mixture of MAR models. We find the solution for both of them using expectation–maximisation (Dempster et al., 1977). The last choice is to run the entire HMM–MAR variational inference procedure a number of times, using pure random initialisation and a low number of variational iterations, so that we would use the one with the lowest free energy as a starting point of an inference run that we will eventually allow to converge. The last strategy can be generalised to a pyramid of runs, where the runs at each level start with the best of a subset of HMM–MAR models from the immediately lower level (which would have been run for a shorter time). We have not explored this last alternative.

The best option depends on the problem at hand and it is difficult to provide a general recommendation. A reasonable possibility is to try all different initialisations, choosing the one with the best free energy. In the reported experiments, we have used the mixture of MAR models, randomly initialised. Although we did not find big differences between the initialisation methods for our data, we are aware that other data sets may be more sensitive to this choice.

## Appendix E Parametric frequency estimation

The autoregression coefficient matrices can be mapped to the frequency domain for frequency *f* as

$$V^{\left(k\right)}\left(f\right)=\sum_{l=1}^{P}W_{l}^{\left(k\right)}e^{-i2\mathrm{\pi flT}}\text{.}$$

Let us denote ${\bar{V}}^{\left(k\right)}\left(f\right)=I-V^{\left(k\right)}\left(f\right)$. Now, for each state *k*, given the noise covariance matrix **Σ**^(*k*)^ and the transfer matrix, $H^{\left(k\right)}\left(f\right)={\bar{V}}^{\left(k\right)}{\left(f\right)}^{-1}$, we can readily estimate the PSD matrix as

(14) $$S^{\left(k\right)}\left(f\right)=H^{\left(k\right)}\left(f\right)\;\Sigma^{{\left(k\right)}^{-1}}\;H^{\left(k\right)}\left(f\right)\text{.}$$

Using ***S***^(*k*)^(*f*), we can compute the coherence between channels *i* and *j* as

$${Coh}_{ij}^{\left(k\right)}\left(f\right)=\frac{S_{ij}^{\left(k\right)}\left(f\right)}{\sqrt{S_{ii}^{\left(k\right)}\left(f\right)S_{jj}^{\left(k\right)}\left(f\right)}}\text{.}$$

Finally, we can obtain the PDC from channel *i* to channel *j* as

$${PDC}_{ij}^{\left(k\right)}\left(f\right)=\frac{\Sigma_{jj}^{{\left(k\right)}^{-1}}{\bar{V}}_{ij}^{\left(k\right)}\left(f\right)}{\sqrt{\sum_{m=1}^{M}\Sigma_{\mathrm{mm}}^{{\left(k\right)}^{-2}}{\left|{\bar{V}}_{im}^{\left(k\right)}\left(f\right)\right|}^{2}}}\text{.}$$

Note that, although these equations are valid per se when using either an exponential lapse or an offset *P*_0_ > 0, they will introduce different levels of resolution at different frequencies and, potentially, miss important information that could lead to misleading spectra. For example, the exponential lapse will result in spectra with more resolution at the lower frequencies than at the higher frequencies. Therefore, if the model has been trained with a non-standard parametrisation, it is recommendable to obtain (once the segmentation is finished) a standard MAR by completing the missing lags, and then use it for the estimation of the frequency information.

## Appendix F Non-parametric PDC

We can use the (statewise) multitaper to obtain ready estimations of PSD and coherence, but we cannot obtain PDC directly because it explicitly needs the autoregression coefficients. We can however reformulate the PDC in a non-parametric way as

$${PDC}_{ij}^{\left(k\right)}\left(f\right)=\frac{\left|J_{ij}^{\left(k\right)}\left(f\right)\right|}{\sqrt{\sum_{m=1}^{M}{\left|J_{im}^{\left(k\right)}\left(f\right)\right|}^{2}}}$$

where ***J***(*f*) = ***H***(*f*)^− 1^ (Jachan et al., 2009). Now, given the expression 5 to compute the PSD matrix, and having computed ***S***^(*k*)^(*f*) via the statewise multitaper from Eq. (14), we can apply the Wilson algorithm on ***S***^(*k*)^(*f*) to obtain a factorisation that approximates ***H***(*f*). The Wilson algorithm was proposed in the applied mathematics literature as an iterative Newton's method to compute an approximate but unique solution of the matrix square root problem. On the downside, it can be computationally costly in high dimensions, which precludes its use within window-based approaches.

## Appendix G Comparison with a stationary MAR model

Considering the high amount of explained variance that a single MAR can achieve on MEG data (in this case higher than 99% for a MAR order of 8), one might question whether a single global MAR model is by itself enough to describe the data sufficiently. Clearly a single MAR cannot find transient patterns of activity. For example, a stationary MAR model does not contain any information about the button press event. As a straightforward demonstration of this, we estimate a stationary MAR model from the MEG ROI time courses, complex enough to explain 99.99% of the variance. Then, we sample a data set from this MAR model (with the same size as the original data set), and run the HMM–MAR inference procedure to see if the inferred time courses reveal something related to the event. As expected the inferred state time courses are random processes with no relation to the event (see Fig. 9).
